# Superoxide Dismutase 3 Deficiency Disrupts the Regulation of Oxidative Stress Caused by Polystyrene Nanoplastics

**DOI:** 10.3390/antiox14111378

**Published:** 2025-11-19

**Authors:** Yugyeong Sim, Jin-Hyoung Kim, Jeong-Soo Lee, Jinyoung Jeong, Hyun-Ju Cho

**Affiliations:** 1 Environmental Disease Research Center, Korea Research Institute of Bioscience and Biotechnology, Daejeon 34141, Republic of Korea; syugg429@kribb.re.kr; 2 KRIBB School, University of Science and Technology, Daejeon 34113, Republic of Korea; jeongsoo@kribb.re.kr; 3 Division of Life Sciences, Korea Polar Research Institute, Incheon 21990, Republic of Korea; kimjh@kopri.re.kr; 4 Polar Science Department, University of Science and Technology, Incheon 21990, Republic of Korea; 5 Microbiome Convergence Research Center, Korea Research Institute of Bioscience and Biotechnology, Daejeon 34141, Republic of Korea

**Keywords:** superoxide dismutase 3, nanoplastics, oxidative stress, cell death, intestinal function and immune response

## Abstract

Nanoplastics have been recognized as emerging pollutants posing potential risks to ecosystems and human health. They are now detected ubiquitously in the environment and even human tissues, where their small size allows for tissue accumulation and cellular penetration. Growing evidence links nanoplastics to oxidative stress, yet the specific contribution of extracellular accumulation to toxicity remains poorly understood. To address this, we used zebrafish, a transparent vertebrate model suitable for toxicological studies, to explore the role of extracellular antioxidant defenses in polystyrene nanoplastic (PSNP)-induced oxidative stress. In particular, we focused on superoxide dismutase 3 (SOD3), which is an enzyme that regulates extracellular reactive oxygen species by catalyzing the detoxification of superoxide radicals. Zebrafish Sod3a is a homolog of human SOD3, preserving conserved metal-binding sites critical for enzymatic function. We established *sod3a* mutant zebrafish and examined their responses following PSNP exposure. In *sod3a* mutant larvae, tissue accumulation of PSNPs was higher than in wild-type (WT), and this was associated with elevated oxidative stress, enhanced cell death, and abnormalities in intestinal function and immune responses. Collectively, these observations reveal the functional importance of SOD3 during PSNP-induced oxidative stress and provide new insight into extracellular antioxidant mechanisms that mitigate PSNP-induced toxicity.

## 1. Introduction

Nanoplastics, which are ultra-fine plastic particles measuring between 1 and 1000 nm in diameter, have emerged as a growing concern for both the environment and public health. These particles are produced as plastics gradually break down under environmental factors such as ultraviolet radiation, mechanical abrasion, heat exposure, and biological processes [1,2]. These nanoplastic particles are widely distributed in aquatic ecosystems, soil, and the atmosphere, and they can enter plants, animals, and humans through various pathways. Due to their small size, nanoplastics can accumulate within animal tissues or penetrate into cells, raising concerns about their long-term risks to human health [3,4]. Recent studies have confirmed the accumulation of MNPs (microplastics and nanoplastics) in human carotid artery plaques and even brain tissue. Notably, brains of patients with dementia showed more than tenfold higher levels of MNP accumulation compared to healthy individuals [5,6,7]. This finding highlights the potential threat of nanoplastics to human health and emphasizes the need for systematic toxicity studies.

Because of their unique properties, such as small size and large surface area, nanoplastics can cross biological barriers and become distributed throughout the body. Specifically, they can associate not only with intracellular structures such as organelles and proteins but also with extracellular matrix (ECM) components and interstitial fluid, thereby disrupting cellular homeostasis and inducing various toxic responses [8,9,10]. Although nanoparticles can penetrate cells, many studies have reported that nanoparticles predominantly accumulate in extracellular spaces, where the ECM acts as a diffusion barrier [11,12]. Therefore, they may persist in the extracellular space for extended periods, potentially altering the structure of the extracellular matrix or interfering with cell-to-cell communication, thereby affecting extracellular signaling processes [13].

The zebrafish has been established as an important model organism widely used for research purposes in various fields, including biomedical sciences, environmental toxicology, and drug development. In particular, the optical transparency and rapid reproduction of zebrafish larvae enable real-time developmental studies and rapid establishment of genetically modified lines for high-throughput screening. Zebrafish have also proven valuable for assessing the accumulation and toxic effects of emerging pollutants, various environmental contaminants, and nanoplastics.

Accumulating evidence from previous studies, including zebrafish models, oxidative stress has been identified as a primary toxic mechanism induced by polystyrene nanoplastics (PSNPs) [14,15,16]. Moreover, oxidative stress plays a pivotal role in the various toxic mechanisms underlying nanoplastic-induced toxicity [17,18,19,20]. This process is triggered by an imbalance between reactive oxygen species (ROS) and the antioxidant defense system, leading to damage to cellular membranes, proteins, and DNA, and ultimately resulting in inflammation and cell death, thereby establishing oxidative stress as a key mechanism under intensive investigation. However, research specifically addressing the biological consequences of nanoplastic accumulation within extracellular spaces remains scarce.

In this work, we proposed a novel hypothesis that the extracellular antioxidant enzyme, superoxide dismutase 3 (SOD3), may be involved in the oxidative stress and toxicity mechanisms induced by nanoplastics, using an in vivo zebrafish model. SOD3 is a critical extracellular antioxidant enzyme that converts superoxide anions (O_2_^−^) to hydrogen peroxide (H_2_O_2_), thereby regulating ROS levels in the extracellular space and interstitial tissues. Zebrafish possess two homologues of human *SOD3*, named *sod3a* and *sod3b*, which share sequence identity with the human enzyme (48.3% and 53.8%, respectively) [21]. Both homologues conserve the essential copper (Cu) and zinc (Zn) binding residues required for enzymatic activity, suggesting that their core functional properties are conserved. Among two homologues, *sod3a* is reported to be expressed in the liver, ovary, and intestine [21]. Given that PSNPs tend to accumulate near the intestinal region, this study focused on *sod3a* and its response to PSNPs. Accordingly, we generated *sod3a* mutant zebrafish to evaluate responses to PSNP exposure, aiming to elucidate the functional role of extracellular SOD3 in nanoplastic-induced oxidative stress and toxicity mechanisms.

## 2. Materials and Methods

### 2.1. Zebrafish Husbandry

Wild-type (WT) AB strain and *sod3a-/-* mutant zebrafish (*Danio rerio*) were maintained under standard zebrafish husbandry conditions in a circulating water system at 28.5 °C with a photoperiod of 14:10 h (light–dark); they were fed Gemma Micro 300 (Skretting, Stavanger, Norway). Embryos were raised in Petri dishes containing 1X E3 egg water (5 mM NaCl, 0.17 mM KCl, 0.33 mM CaCl_2_, 0.33 mM MgSO_4_) at 28.5 °C in a temperature-controlled incubator.

### 2.2. Generation of a Zebrafish sod3a Mutant Line

The *sod3a* mutant zebrafish line was generated using CRISPR/Cas9-based genome editing. A gene-specific single-guide RNA (sgRNA) targeting exon 2 of the *sod3a* gene (sequence: CAATACGGAGACCTCAGTCA) was designed using the CHOPCHOP tool version 2 (https://chopchop.cbu.uib.no, accessed on 17 April 2025). The sgRNA was cloned into the DR274 plasmid, which was then linearized for in vitro transcription using the MAXIscript kit (Invitrogen, Carlsbad, CA, USA). Approximately 1–2 nL of a solution containing 30 ng/μL sgRNA and 500 ng/μL Cas9 protein (NEB, lpswich, MA, USA #M0646T) was microinjected into one-cell stage zebrafish embryos. The injected embryos were raised to adulthood (F0), and genomic DNA was extracted from fin clips or pools of larvae. The target region was amplified by PCR and subjected to Sanger sequencing to identify mutations. Primer sequences used for PCR amplification of the target region are provided in Table S1.

### 2.3. In Situ Hybridization and Gene Expression Analysis

Whole-mount in situ hybridization (WISH) was performed to assess the expression pattern of *sod3a*, as described in a previous study on zebrafish larvae [22]. Primer sequences used for probe synthesis are listed in Table S1. For quantitative analysis of transcription levels of genes involved in immune responses in zebrafish embryos, total RNA was isolated using TRIzol reagent solution (15596-026, Invitrogen, Carlsbad, CA, USA) according to the manufacturer’s instructions. RNA purification was performed using the Direct-zol RNA Miniprep Kit (Zymo Research, Irvine, CA, USA). First-strand cDNA synthesis was carried out using the SuperScript III First-Strand Synthesis System (Invitrogen). Gene-specific PCR was performed using cDNA templates, with primer sequences listed in Table S1.

### 2.4. Fluorescent Quantification of PSNP Accumulation

In this study, 50 nm polystyrene nanoplastics, both non-fluorescent (Cat. 08691) and green-fluorescent labeled (Cat. 17149), were purchased from Polysciences, Inc. (Warrington, PA, USA) as 2.6% solid suspensions. The PSNPs were dispersed in distilled water and egg water prior to exposure experiments, and their size distribution was analyzed using a nanoparticle tracking analyzer (ZETAVIEW, PARTICLE METRIX, Meerbusch, Germany). To evaluate PSNP accumulation, two complementary approaches were used: microscopy-based fluorescence analysis and total fluorescence quantification. For microscopy-based fluorescence analysis, 4 dpf zebrafish larvae (n = 15 per group) were exposed to 5 mL treatment solutions containing 0, 1, 10, or 50 μg/mL PSNPs and incubated for 3 days at 28.5 °C. On the final day of exposure at 7 dpf, the larvae were rinsed with fresh 1X E3 egg water and anesthetized with tricaine (MS222, Supelco, St. Louis, MO, USA). PSNP biodistribution was visualized using a stereoscopic microscope equipped with a filter set (excitation: 480 nm, emission: 535 nm) (SMZ18, Nikon, Japan), and fluorescence intensity in the intestinal regions was quantified using ImageJ software 1.52a (NIH, Bethesda, MD, USA). For total fluorescence quantification, larvae (n = 10 per group) were homogenized in 200 μL of distilled water and centrifuged for 30 s at 5000 rpm (Smart 13; Hanil, Gimpo, Republic of Korea) to spin down the tissue. The fluorescence intensity of the supernatant was measured using a microplate reader (Cytation 5, BioTek, Winooski, VT, USA) with excitation at 441 nm and emission at 486 nm. The PSNP amount in the larvae was calculated based on fluorescence values using a standard curve. All experiments were performed in triplicate.

### 2.5. ROS Detection (CM-H_2_DCFDA Assay)

Zebrafish larvae were incubated with 10 μM 2′,7′-dichlorodihydrofluorescein diacetate (CM-H_2_DCFDA, C6827, Invitrogen, MA, USA) for 1 h at 28.5 °C in the dark. Following incubation, the larvae were rinsed with fresh egg water and anesthetized with tricaine. ROS signals were visualized using a fluorescence microscope (SMZ18, Nikon, Japan) equipped with a filter set (excitation: 480 nm; emission: 535 nm). Fluorescence intensity in the intestinal region was quantified using ImageJ software 1.52a (NIH, Bethesda, MD, USA). Fifteen larvae were analyzed per group, and all experiments were performed in triplicate.

### 2.6. Hydrogen Peroxide Detection (Amplex Red Assay)

Hydrogen peroxide (H_2_O_2_) levels released from zebrafish larvae were quantified using the Amplex^®^ Red Hydrogen Peroxide/Peroxidase Assay Kit (Invitrogen, A22188, MA, USA) according to a modified protocol [23]. Zebrafish larvae (7 dpf, n = 30 per group) were washed three times with PBS and homogenized in 100 μL of ice-cold reaction buffer using a micro pestle. The homogenate was centrifuged (3000 rpm, 1 min), and 50 μL of the supernatant was transferred to a black 96-well microplate. The reaction mixture was freshly prepared by combining Amplex^®^ Red (final 50 μM), horseradish peroxidase (HRP; final 0.1 U/mL), and 1× reaction buffer. A standard curve was generated separately using serial dilutions of a 20 mM H_2_O_2_ stock to produce final concentrations of 0, 0.5, 1, 2, 3, and 4 μM (after 1:1 mixing with Amplex^®^ Red mix). For each standard or zebrafish sample, 50 μL of solution was mixed with 50 μL of the Amplex^®^ Red reaction mix to a final volume of 100 μL. Samples and standards were incubated at room temperature (25 °C) or 28.5 °C for 60 min in the dark. Fluorescence was measured using a microplate reader (excitation: 540 ± 20 nm; emission: 590 ± 20 nm) (Cytation 5, Bio Tek, Winusky, VT, USA). Raw fluorescence values were background-subtracted using the absence of H_2_O_2_ as the baseline (rRFU = RFU_sample_ − RFU_blank_), and H_2_O_2_ concentrations were interpolated from a standard curve constructed via linear fit (Origin 2020b). The final amount of H_2_O_2_ was expressed as pmol per 30 larvae or as fold change compared with the control group.

### 2.7. Acridine Orange Staining

To assess cell death, zebrafish larvae were stained with acridine orange (10 μg/mL) (A9231; Sigma, St. Louis, MO, USA) for 30 min at 28.5 °C in the dark. The larvae were then rinsed with fresh 1X E3 egg water and anesthetized with tricaine to measure the green fluorescence signal of cell death using a fluorescence microscope (SMZ18, Nikon, Japan) equipped with a filter set (excitation: 480 nm; emission: 535 nm). The intensity of the fluorescent signal was quantified using ImageJ software 1.52a (NIH, Bethesda, MD, USA). Fifteen larvae were analyzed per group, and all experiments were performed in triplicate.

### 2.8. Zebrafish Intestinal Motility

Zebrafish larvae (n = 10 per group) were exposed to 50 μg/mL PSNPs for 3 days. Following exposure, the larvae were rinsed with fresh EW and anesthetized in 0.04% tricaine for approximately 1 min. The anesthetized larvae were embedded in 2.5% methylcellulose and positioned for imaging. A 5-min video of the entire intestinal region was recorded using a stereoscopic microscope (SMZ18, Nikon, Japan). Intestinal motility in zebrafish larvae was characterized by two distinct directions: anterograde movement (from the middle intestine toward the anus) and retrograde movement (from the middle intestine toward the anterior intestine). Intestinal motility was quantified based on two parameters: contraction frequency (the number of contractions per minute) and contraction interval (the time between two consecutive contractions).

### 2.9. Statistical Analysis

The statistical analysis was performed using Origin 2020b software (OriginLab Corporation, Northampton, MA, USA). One-way analysis of variance (ANOVA) was conducted, followed by Tukey’s post hoc test for multiple comparisons. Differences were considered statistically significant at *p* < 0.05.

## 3. Results

### 3.1. Spatial Expression of sod3a and Its Association with PSNP Accumulation

To study PSNP-induced oxidative stress toxicity, we generated *sod3a* gene mutants using the CRISPR-Cas9 system. As a result, *sod3a-/-* zebrafish carrying a 22 bp insertion and a 205 bp deletion in exon 2 within the *sod3a* locus were obtained. Amino acid sequence alignment revealed that the mutated region corresponds to a conserved enzymatic domain of human SOD3, with a substantial portion deleted (Figure S1). The *sod3a-/-* zebrafish developed normally from embryonic stages to adulthood, exhibited no obvious morphological abnormalities, and retained normal reproductive capacity.

**Figure 1:**
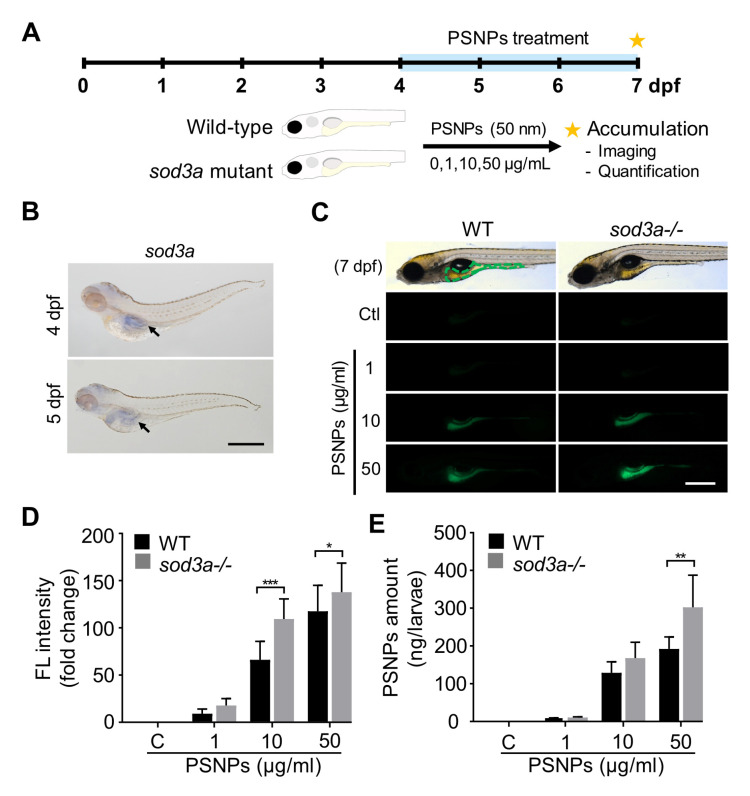
PSNP accumulation is altered in WT and *sod3a-/-*. (**A**) The experimental scheme illustrates the analysis of PSNP accumulation in zebrafish larvae at 7 dpf. (**B**) Whole-mount in situ hybridization (WISH) images show *sod3a* expression in the intestinal region (black arrow) of WT. (**C**) Fluorescent images display zebrafish larvae exposed to PSNPs for 72 hpi, with green dotted lines indicating the intestinal region. (**D**) Microscopy-based fluorescence analysis of PSNPs in the intestinal region and (**E**) the total amount of PSNPs in the whole body of zebrafish larvae at 7 dpf is quantified. Data are presented as mean ± standard error of the mean, and significant differences between two groups (Tukey’s test) are represented as * *p* < 0.05, ** *p* < 0.01, *** *p* < 0.001. Scale bar: 500 μm.

To investigate the potential involvement of *sod3a* in the toxic response induced by PSNP exposure, we designed an experimental timeline, as seen in Figure 1A. The PSNPs used in this study had an average size of 50 nm, as shown in Figure S2. Before PSNP exposure, we examined the spatial expression patterns of *sod3a* in zebrafish larvae using whole-mount in situ hybridization (WISH). As a result, *sod3a* was strongly expressed in the intestinal and hepatic regions during 4–5 dpf (days post-fertilization) (Figure 1B).

To evaluate the possible role of *sod3a* in the uptake, metabolism, or excretion of PSNPs, we first compared the PSNP accumulation patterns between wild-type (WT) and *sod3a-/-* zebrafish larvae. To determine whether there were differences in PSNP accumulation between WT and *sod3a-/-* larvae, we exposed zebrafish larvae with 1, 10, and 50 μg/mL PSNPs from 4 to 7 dpf and assessed PSNP accumulation. Based on the total PSNP amount by whole larvae, accumulation in WT larvae increased until 24 h at all concentrations tested (1, 10, and 50 μg/mL), after which it reached saturation and showed no further increase up to 72 h (Figure S3, black line). In contrast, in *sod3a-/-* larvae, accumulation of PSNPs increased continuously up to 72 h at 10 and 50 μg/mL, whereas at 1 μg/mL, no significant difference was observed (Figure S3, red line).

To facilitate a precise comparison, we exposed 4 dpf larvae to PSNPs for 3 days and analyzed accumulation at 7 dpf using two complementary approaches: microscopy-based fluorescence analysis and total amount quantification based on PSNP standard curve. The former allowed assessment of PSNP biodistribution in specific regions by measuring fluorescence intensity, while the latter provided quantitative information on total PSNP accumulation in the whole body. To obtain accurate accumulation profiles, we integrated both methods. Microscopy-based fluorescence analysis revealed that PSNPs predominantly accumulated in the intestinal bulb in both WT and *sod3a-/-* (Figure 1C). Based on microscopic analysis, the mutants showed significantly higher accumulation than WT, with 1.7-fold and 1.2-fold increases at 10 and 50 μg/mL, respectively (Figure 1D). Similarly, the quantification analysis of PSNPs in a whole body showed a significant 1.6-fold increase in *sod3a-/-* compared with WT at 50 μg/mL (Figure 1E). These analyses demonstrated that PSNP accumulation was elevated in *sod3a-/-* compared to WT, with the most significant differences in both fluorescence intensity and total amount quantification being observed at 50 μg/mL. Taken together, our results indicate that *sod3a* plays a critical role in regulating PSNP accumulation, particularly under higher-exposure conditions, highlighting its potential involvement in modulating nanoplastic biodistribution and retention in vivo.

### 3.2. Assessment of PSNP-Induced Oxidative Stress

**Figure 2:**
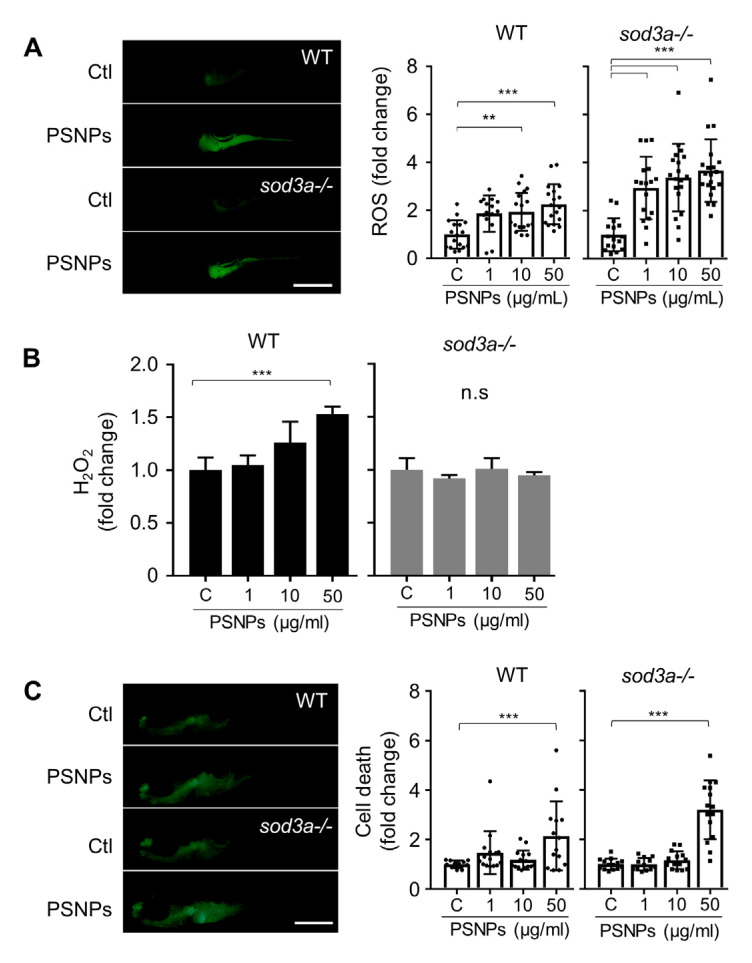
ROS generation and cell death are examined in zebrafish larvae exposed to PSNPs. (**A**) Fold changes and fluorescent images show ROS generation after 72 hpi exposure, with the images corresponding to larvae exposed to 50 μg/mL PSNPs. (**B**) Hydrogen peroxide (H_2_O_2_) levels are measured in whole larvae, and (**C**) cell death detected by acridine orange (AO) is observed in the intestinal region following PSNP exposure (50 μg/mL). Data are presented as mean ± standard error of the mean, and significant differences between control (Tukey’s test) are represented as ** *p* < 0.01, *** *p* < 0.001. Scale bar: 500 μm. n.s. indicates no significant.

To evaluate how SOD3 modulates PSNP-induced oxidative responses, we compared oxidative stress levels between WT and *sod3a-/-* following exposure to PSNPs. WT and *sod3a-/-* were treated with 1, 10, and 50 μg/mL PSNPs, and total ROS levels were assessed using the CM-H_2_DCFDA assay (Figure 2A). In WT larvae, exposure to 1 μg/mL PSNPs did not significantly increase ROS levels, whereas 10 and 50 μg/mL PSNP exposure led to significant increases of 1.94-fold and 2.26-fold, respectively. In contrast, *sod3a-/-* showed a significant 2.94-fold increase in ROS levels, even at 1 μg/mL PSNPs, with further increases to 3.38-fold and 3.67-fold at 10 and 50 μg/mL PSNPs, respectively.

To gain a more detailed understanding of the oxidative stress response, we measured H_2_O_2_ levels, a central intermediate in ROS detoxification pathways and an important indicator in intracellular signaling [24,25]. In WT larvae, exposure to 50 μg/mL PSNPs significantly increased H_2_O_2_ levels by 1.5-fold, as determined using the Amplex Red assay. In contrast, no significant change in H_2_O_2_ levels was observed in *sod3a-/-* following PSNP exposure (Figure 2B). That there was no change in H_2_O_2_ levels in *sod3a-/-* may be attributed to the absence of Sod3a-mediated conversion of superoxide radicals to H_2_O_2_, suggesting impaired ROS detoxification in these mutants.

### 3.3. Cell Death Comparison PSNP Accumulation

Oxidative stress is known as one of the major mechanisms that can cause cellular damage, leading to cytotoxicity and cell death. Therefore, we aimed to evaluate the association between oxidative stress and cell death following PSNP exposure. Cell death was assessed using acridine orange (AO) staining, which was particularly conducted in the intestinal region where PSNP accumulation and elevated ROS levels were observed (Figure 2C). As a result, an increase in the intensity of AO staining was detected in both WT and *sod3a-/-* groups treated with PSNPs at a concentration of 50 µg/mL, suggesting enhanced cell death due to PSNP exposure in that region. Furthermore, the intensity of AO staining in *sod3a-/-* was about 3.2 times higher than in the control group. This increase was approximately 52.4% greater than the 2.1-fold increase seen in WT, indicating that cell death was more pronounced in *sod3a-/-* compared to WT.

### 3.4. Differential Immune Responses to PSNPs

**Figure 3:**
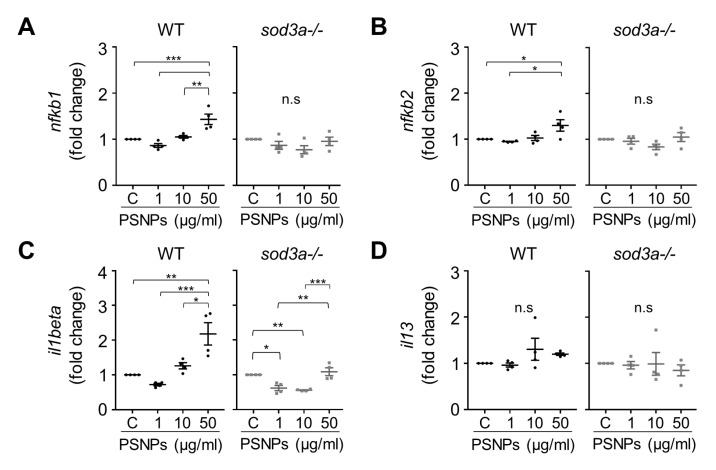
The immune response to PSNP exposure is examined through gene expression of (**A**) *nfkb1*, (**B**) *nfkb2*, (**C**) *il1beta*, and (**D**) *il13* in WT and *sod3a-/-*. Data are presented as mean ± standard error of the mean, and significant differences between control (Tukey’s test) are represented as * *p* < 0.05, ** *p* < 0.01, *** *p* < 0.001. n.s. indicates no significant.

Since oxidative stress resulting from ROS accumulation is closely associated with the activation and regulation of the immune system, analyzing immune responses provides important information for understanding the mechanisms of toxicity. To investigate how PSNP accumulation and oxidative stress may affect the immune response, we examined the transcription patterns of immune-related genes in WT and *sod3a-/-* under PSNP exposure. We analyzed *il1beta* as a representative pro-inflammatory cytokine, along with *nfkb1* and *nfkb2*, which are members of the NF-κB family and also known transcriptional targets of this pathway [26]. In addition, *il13* was included to evaluate the effects of PSNP exposure on both pro-inflammatory and Th2-type cytokine expression. In WT, exposure to 50 µg/mL PSNPs resulted in fold increases of 1.43, 1.30, and 2.18 for *nfkb1*, *nfkb2*, and *il1beta*, respectively, compared to the control (Figure 3A–C). However, in *sod3a-/-*, this increase in immune response was not significant at 50 µg/mL PSNPs, and *il1beta* expression was, in fact, reduced, with fold changes of 0.62 and 0.56 at 1 and 10 µg/mL PSNPs, respectively. Furthermore, no significant changes in *il13* gene expression were observed in either WT or *sod3a-/-* (Figure 3D). These results indicate that, in WT, PSNP accumulation leads to NF-κB-related pro-inflammatory immune responses, whereas *sod3a-/-* shows little to no immune response or exhibits an abnormal pattern characterized by reduced immune activation.

### 3.5. Differential Intestinal Motility Responses to PSNP Exposure

**Figure 4:**
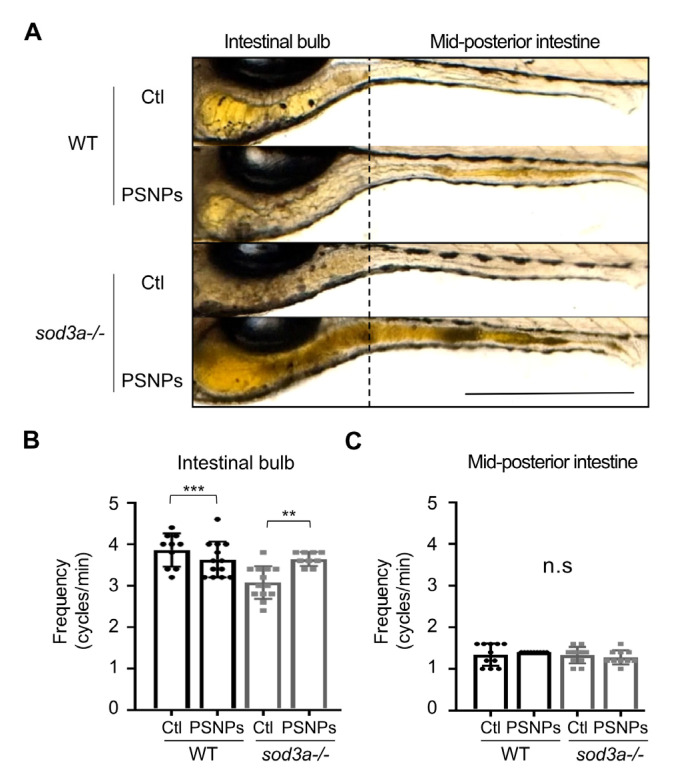
Intestinal motility is examined in zebrafish larvae exposed to PSNPs (50 μg/mL) for 72 hpi. (**A**) Two parts of the intestine are indicated in zebrafish larvae. (**B**) The frequency of retrograde contractions is measured in the intestinal bulb, and (**C**) the frequency of anterograde contractions is measured in the mid-posterior intestine. Data are presented as mean ± standard error of the mean, and significant differences between two groups (Tukey’s test) are represented as ** *p* < 0.01, *** *p* < 0.001. Scale bar: 500 μm. n.s. indicates no significant.

We assessed intestinal motility patterns by functional region to determine the association between PSNPs’ adverse effects and intestinal motility. In the anterior region, including the intestinal bulb, retrograde movements occur primarily to regulate retention and prevent reflux of luminal contents, while anterograde movements are observed in the mid-posterior intestine to facilitate excretion [27,28,29]. Accordingly, we divided the zebrafish larvae intestine into regions corresponding to retrograde and anterograde movements and analyzed the changes in intestinal motility after PSNP exposure in both WT and *sod3a-/-* (Figure 4A). First, we examined intestinal motility under PSNP exposure at 50 µg/mL in WT, as this concentration showed the greatest differences in PSNP accumulation, cell death, and ROS levels between WT and *sod3a-/-*. However, no significant differences were found in either retrograde or anterograde movements compared to the control, indicating that PSNPs themselves do not substantially affect intestinal motility in WT (Figure 4B).

Next, to compare the intestinal motility responses of WT and *sod3a-/-* to PSNP exposure, we examined their basal intestinal motility under control conditions as a preliminary step. As a result, we found that while anterograde movement was comparable between WT and *sod3a-/-*, retrograde movement was reduced in *sod3a-/*-. When exposed to PSNPs, *sod3a-/-* at 50 µg/mL showed a significant increase in retrograde movement. However, anterograde movement remained unaffected (Video S1). These results suggest that, in *sod3a-/-*, retrograde motility is impaired under basal conditions but responds sensitively to PSNP-induced stimulation.

## 4. Discussion

**Figure 5:**
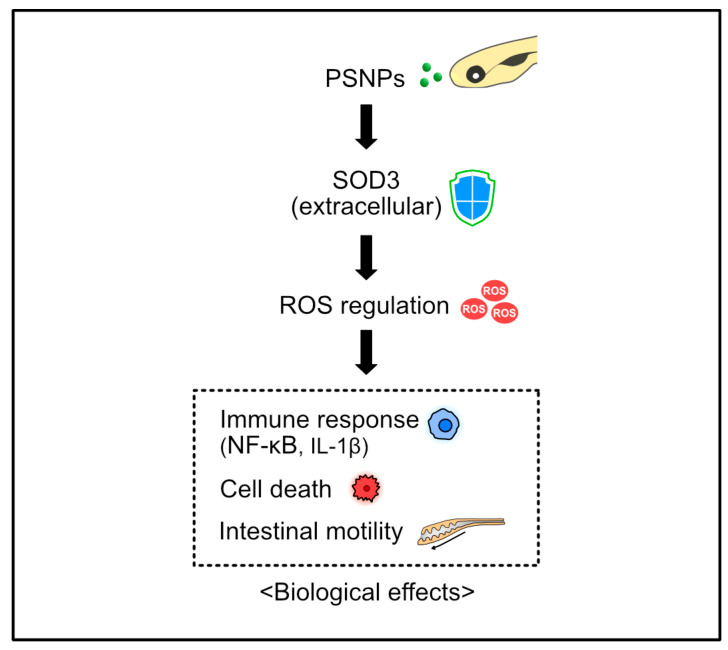
The schematic illustrates SOD3-mediated extracellular ROS regulation and its associated biological effects upon PSNP exposure. Extracellular SOD3 modulates ROS levels, influencing immune responses, cell death and intestinal motility.

In this experiment, WT and *sod3a-/-* zebrafish larvae were exposed to PSNPs under identical conditions to comprehensively analyze the extent and distribution of PSNP accumulation, as well as associated physiological and immunological toxic responses. As a result, the *sod3a*-/- larvae exhibited greater accumulation of PSNPs in the intestinal region compared to WT, which was accompanied by increased oxidative stress responses and cell death, along with abnormal intestinal function and immune responses. These findings suggest that the extracellular ROS elimination mechanism plays a critical protective role in regulating PSNP-induced toxicity. Furthermore, this study provides important insight into the function of the extracellular antioxidant defense system and the biological disruption caused by nanoplastic exposure (Figure 5).

The previous studies have primarily focused on intracellular oxidative stress mechanisms, reporting changes in the activity or gene expression of antioxidant enzymes such as SOD1, SOD2, and catalase in response to PSNP exposure. However, studies addressing SOD3, which is involved in extracellular oxidative stress signaling, remain extremely limited (Table 1). SOD3 is a crucial antioxidant enzyme that plays a key role in maintaining extracellular redox homeostasis. Notably, SOD3 is thought to represent an evolutionarily conserved system for regulating ROS. For instance, an increased copy number of the *sod3* gene has been observed in the genome of the Antarctic blackfin icefish, suggesting that SOD3 likely plays an essential protective role in the extreme Antarctic environment and may have an evolutionarily conserved function in oxidative stress regulation [30].

PSNPs can enter the body through the respiratory or gastrointestinal tracts. After entry, they may remain in the extracellular space or penetrate into cells, interacting with the ECM in various ways during these processes. Such interactions can disrupt ECM-based cell signaling pathways, potentially leading to diverse toxic responses. Thus, we examined the possibility that PSNPs may remain in the extracellular space and influence oxidative stress signaling through extracellular antioxidant pathways such as SOD3. In line with this, recent studies have shown that mice exposed to airborne PSNPs exhibited impaired cardiac function and histopathological changes, such as myocardial hypertrophy and fibrosis. These alterations were found to be mediated through interactions between ECM receptors and the PI3K/AKT/BCL-2 signaling pathway [39]. Additionally, transcriptome analysis using human induced pluripotent stem cells (hiPSCs) revealed significant changes in the expression of ECM-related gene sets following PSNP exposure [40]. Furthermore, molecular dynamics (MD) simulation studies have demonstrated that PSNPs can directly interact with the lipid bilayer of the cell membrane, binding to membrane components or inducing structural alterations, thereby facilitating their cellular entry [41]. Collectively, these findings suggest that PSNPs can disrupt the interactions between ECM components and cell surface receptors, which act as mediators of intercellular signaling, ultimately leading to the collapse of extracellular signaling systems and resulting in various toxic effects.

Additionally, SOD3 serves as an extracellular antioxidant enzyme that plays a crucial role in protecting ECM components such as collagen I and elastin from oxidative damage. Deficiency of SOD3 has been reported to weaken the structural stability of the ECM and increase inflammatory cell infiltration and tissue permeability due to oxidative injury [42,43,44]. In addition, disruption of extracellular redox balance may affect immune responses and cellular functions [45]. Therefore, SOD3 deficiency may facilitate PSNP penetration and retention by promoting ECM degradation and epithelial barrier disruption, which could account for the increased accumulation of PSNPs observed in *sod3a-/-* larvae. However, future investigations will help to elucidate the precise molecular mechanisms underlying this effect.

Since oxidative stress is closely associated with immune regulation, we next investigated whether PSNP exposure in WT zebrafish activates inflammatory signaling pathways. In WT exposed to polystyrene nanoplastics (PSNPs), activation of the NF-κB signaling pathway was observed, accompanied by increased mRNA expression of pro-inflammatory cytokines such as *il1beta*. In contrast, the expression of *il13*, a cytokine involved in Th2 immune responses, did not show significant changes upon PSNP exposure, indicating that the inflammatory response induced by PSNPs is governed by a specific NF-κB pathway. This is further supported by a recent study showing that administration of 20 nm PSNPs in mice activated NF-κB signaling in liver tissue and upregulated pro-inflammatory cytokines such as *il1beta* [46]. These findings are in agreement with previous reports demonstrating that NF-κB signaling plays a central role in microplastic-induced inflammatory responses [47,48,49]. However, in *sod3a-/-*, immune responses via the NF-κB pathway were not induced even after PSNP exposure, and no change in *il13* expression was observed. These results suggest that the normal immune response is either suppressed or dysregulated in *sod3a-/-* individuals. This may be due to impaired detoxification of ROS, as the loss of Sod3a likely disrupts the conversion of superoxide into hydrogen peroxide (H_2_O_2_). Moreover, excessive accumulation of ROS upon PSNP exposure in *sod3a-/-* larvae may lead to compromised ROS defense systems, resulting in abnormal immune responses and increased cell death. Such excessive ROS accumulation is likely to attenuate immune responses. Previous studies have reported that sustained oxidative stress can inhibit NF-κB activity through mechanisms such as IKK inactivation or proteasome impairment, thereby reducing cytokine expression [50]. In addition, several studies have also reported that elevated oxidative stress can impair immune responses by causing cellular damage, protein oxidation, and apoptosis, which lead to decreased cytokine production and immune function [51,52].

Taken together, our findings suggest that SOD3 acts as a key regulator of immune responses and ROS metabolism under PSNP exposure and that its deficiency leads to abnormal ROS accumulation, dysregulation of immune responses, and accelerated accumulation of PSNPs in the body. Therefore, SOD3 may serve as an important biological marker for host defense mechanisms against PSNP-induced toxicity and could be considered a valuable target for future nanomaterial toxicity assessment and safety research.

However, this study is limited by examining only the mutation of a single gene, *sod3a*, and thus does not fully account for potential interactions with other extracellular antioxidant pathways, such as *sod3b*. In addition, since *sod3a-/-* alone can lead to altered ROS levels and impaired intestinal motility, a more precise approach is required to clearly distinguish PSNP-induced effects from baseline phenotypes associated with *sod3a* mutation. Furthermore, investigation of the downstream pathways related to oxidative stress and apoptosis will be necessary to achieve a more precise mechanistic understanding. Comparative studies examining biological responses to nanoplastics with varying sizes and surface properties, along with network analyses of other antioxidant factors, will be essential for achieving a more systematic understanding.

## 5. Conclusions

We demonstrated that SOD3 deficiency leads to excessive nanoplastic accumulation, increased reactive oxygen species production, and various physiological disorders, including impaired intestinal motility and immune function. These results highlight the pivotal role of SOD3 in extracellular redox homeostasis, particle clearance, and immune regulation. Importantly, these insights were achieved through an in vivo model, which highlights the strength of genetic approaches in elucidating physiological mechanisms and offers perspectives that extend beyond the intracellular focus of previous antioxidant studies.

## Figures and Tables

**Table 1:** Summary of previous studies on the impact of MNPs on SOD activity (focused on SOD1, SOD2, and SOD3).

Species	MNPs Type	MNPs Size	MNPs Concentration	Exposure Time	Oxidative Unbalance	Reference
Mouse hepatocytes	PS	0.5, 5 μm	10 mg/L	90 d	SOD2↓	[31]
A549 cell line	PS	0.8 μm	10~500 μg/mL	24 h	SOD1↓, SOD2↓	[32]
*Caenorhabditis* *elegans*	PS	50, 500 nm	1, 10, 15 μg/L	L1 to adult	*sod-2*↑, *sod-3*↑ *	[33]
Zebrafish larvae (*Danio rerio*)	LDPE	<17.6 μm	5, 50, 500 mg/L	10, 20 d	No difference in *sod1*	[34]
	PS	50 nm	1 mg/L	96 h	*sod1*↓, *sod2*↓	[35]
	PS	30 nm	0.1, 0.5, 3 μg/mL	120 h	SOD1↑, SOD2↑	[36]
	Photoaged PS	10 μm	0, 0.1, 1, 10, 100 μg/L	120 h	*sod1*↓	[37]

* In *C. elegans*, five SOD-encoding genes have been reported: *sod-1* and *sod-5*, which produce cytoplasmic Cu/ZnSODs; *sod-2* and *sod-3*, encoding mitochondrial MnSODs; and *sod-4*, responsible for an extracellular Cu/ZnSOD [38].

## Data Availability

The original contributions presented in this study are included in the article/Supplementary Materials. Further inquiries can be directed to the corresponding authors.

## Supplementary Materials

The following supporting information can be downloaded at: https://www.mdpi.com/article/10.3390/antiox14111378/s1, Figure S1: Analyses of zebrafish *sod3a* show (a) WISH negative control, (b) gene structure with insertion/deletion sites, and (c) sequence alignment of the 96–193 bp region of the gene with human SOD3, performed using CLUSTAL multiple sequence alignment (MUSCLE v3.8); Figure S2: Size distribution of PSNPs in (a) distilled water and (b) egg water using nanoparticle tracking analyzer (ZETAVIEW, PARTICLE METRIX, Meerbusch, Germany); Figure S3: PS amounts in the whole body of WT and *sod3a-/-* zebrafish larvae exposed to PSNPs (50 μg/mL) from 4 to 7 dpf, measured at 24, 48, and 72 hpi; Table S1: Primer sequences used for WISH probe and qRT-PCR; Video S1: Intestinal motility of larvae exposed by PSNPs (50 μg/mL) for 72 hpi.

## Abbreviations

The following abbreviations are used in this manuscript:

ANOVA	One-way analysis of variance
AO	Acridine orange
Cu	Copper
ECM	Extracellular matrix
dpf	days of post fertilization
H_2_O_2_	Hydrogen peroxide
hpi	hours of post incubation
HRP	Horseradish peroxidase
MD	Molecular dynamics
MNPs	Microplastics and nanoplastics
O_2_^−^	Superoxide anions
PSNPs	Polystyrene nanoplastics
ROS	Reactive oxygen species
sgRNA	single-guide RNA
SOD3	Superoxide dismutase 3
WT	Wild-type
WISH	Whole-mount in situ hybridization
Zn	Zinc
