# Supplementary material for: Superoxide Dismutase 3 Deficiency Disrupts the Regulation of Oxidative Stress Caused by Polystyrene Nanoplastics
Source: Antioxidants (Basel). 2025 Nov 19;14(11):1378. doi: 10.3390/antiox14111378 (PMC12649132; doi:10.3390/antiox14111378)
Supplement: Supplementary file 1 [file antioxidants-14-01378-s001.zip › antioxidants-3967084-supplementary.pdf]

Supporting Information

Superoxide Dismutase 3 deficiency disrupts the regulation of oxidative stress caused by polystyrene nanoplastics

Yugyeong Sim [1,2], Jin-Hyoung Kim [3,4], Jeong-Soo Lee [2,5], Jinyoung Jeong [1,2,*] and Hyun-Ju Cho [5,*]

1 Environmental Disease Research Center, Korea Research Institute of Bioscience and Biotechnology, Daejeon 34141, Republic of Korea; syugg429@kribb.re.kr

2 KRIBB School, University of Science and Technology, Daejeon 34113, Republic of Korea; jeongsoo@kribb.re.kr

3 Division of Life Sciences, Korea Polar Research Institute, Incheon 21990, Republic of Korea; kimjh@kopri.re.kr

4 Polar Science, University of Science and Technology, Incheon 21990, Republic of Korea

5 Microbiome Convergence Research Center, Korea Research Institute of Bioscience and Biotechnology, Daejeon 34141, Republic of Korea

* Correspondence: jyjeong@kribb.re.kr (J.J.); alleles@kribb.re.kr (H.-J.C.)

Contents

Table S1. Primer sequences used for WISH probe and qRT-PCR.

Figure S1. Analyses of zebrafish *sod3a* show (a) WISH negative control, (b) gene structure with insertion/deletion sites, and (c) sequence alignment of the 96–193 bp region of the gene with human SOD3, performed using CLUSTAL multiple sequence alignment (MUSCLE v3.8).

Figure S2. Size distribution of PSNPs in (a) distilled water and (b) egg water using nanoparticle tracking analysis (ZETAVIEW, PARTICLE METRIX, Meerbusch, Germany).

Figure S3. PSNPs amounts in the whole body of WT and *sod3a*-/- zebrafish larvae exposed to PSNPs (50 μg/mL) from 4 to 7 dpf, measured at 24, 48, and 72 hpi.

Video S1. Gut motility of larvae exposed by PSNPs (50 μg/mL) for 72 hpi.

Table S1. Primer sequences used for WISH probe and qRT-PCR.

Primers sequence for in situ hybridization probe synthesis

Gene	Forward (5′ – 3′)	Reverse (3′ – 5′)	Reference
sod3a	cgaagctccaaaaccggaagtg	tctcttcctcctccacgtgt	FJ807962.1

Primer sequences for qRT-PCR

nfkb1	cgcaagtcctacccacaagt	accagactgtgagcgtgaag	[1]
nfkb2	catatgtcccacacaatcaagac	agccaccataatgatctggaa	[1]
il1b	gaacagaatgaagcacatcaaacc	acggcactgaatccaccac	NM_212844
il13	gtcaggctgaggaggagatg	agcagcgtgactcctgatct	NM_001199905
eef1a1l1	ctggttcaagggatggaaga	cacacgacccacaggtacag	NM_131263

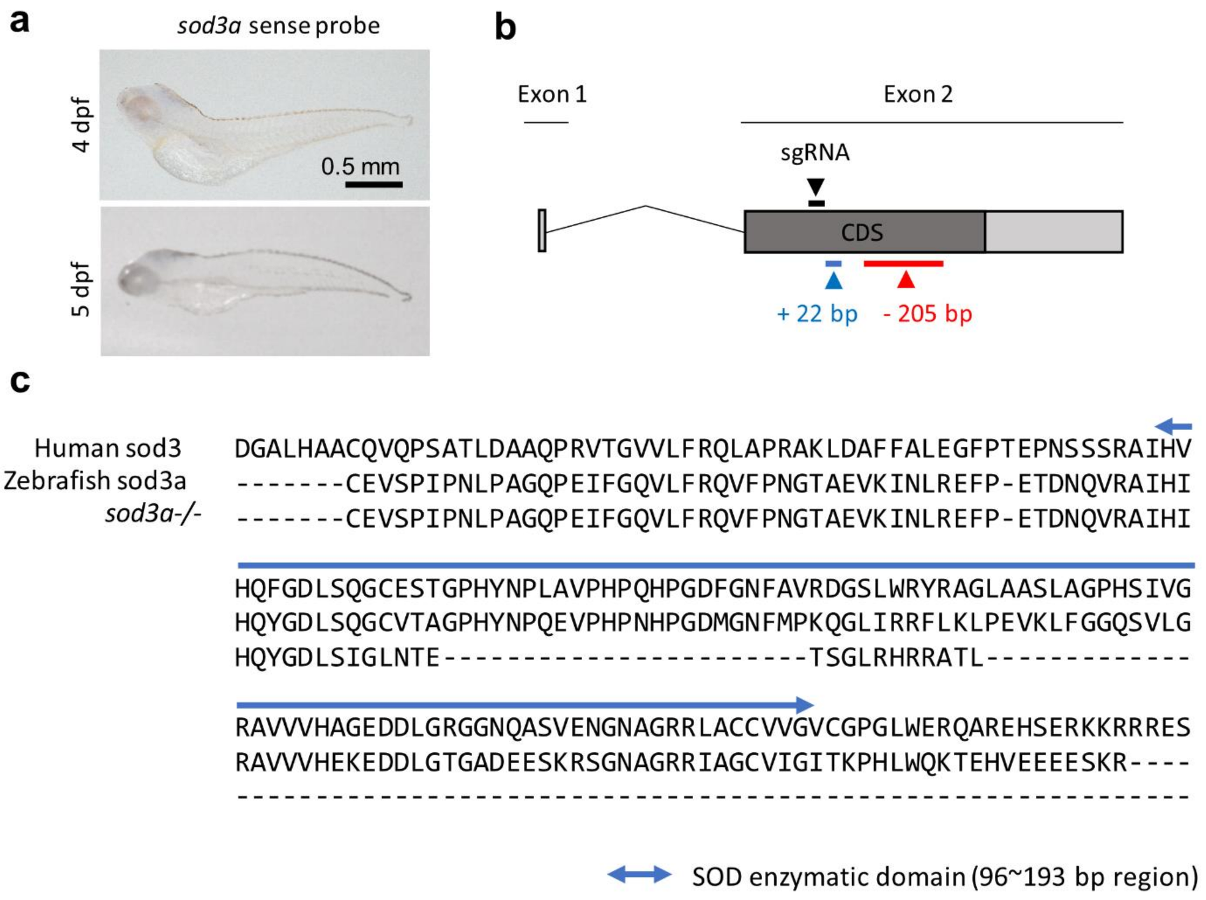

Fig. S1. Analyses of zebrafish *sod3a* show (a) WISH negative control, (b) gene structure with insertion/deletion sites, and (c) sequence alignment of the 96–193 bp region of the gene with human SOD3, performed using CLUSTAL multiple sequence alignment (MUSCLE v3.8).

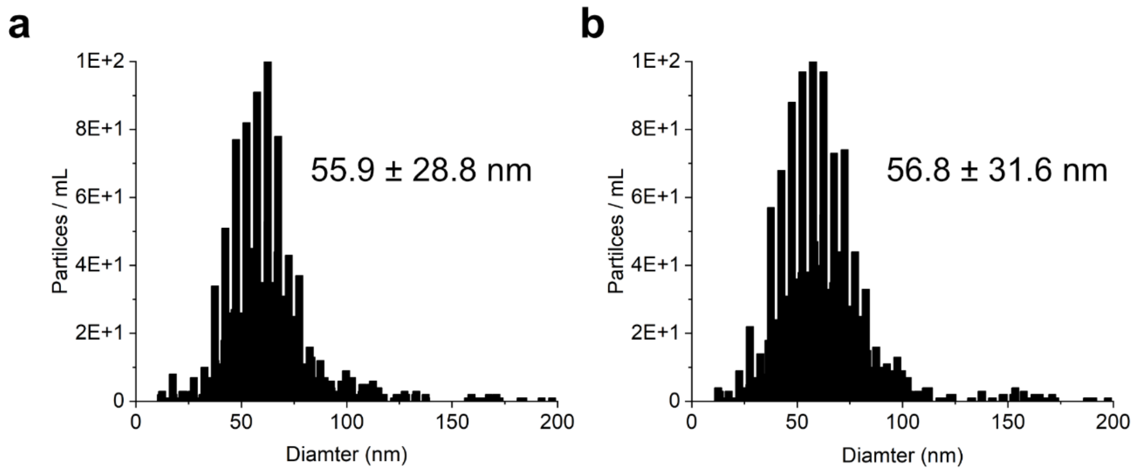

Fig. S2. Size distribution of PSNPs in (a) distilled water and (b) egg water using nanoparticle tracking analysis (ZETAVIEW, PARTICLE METRIX, Meerbusch, Germany).

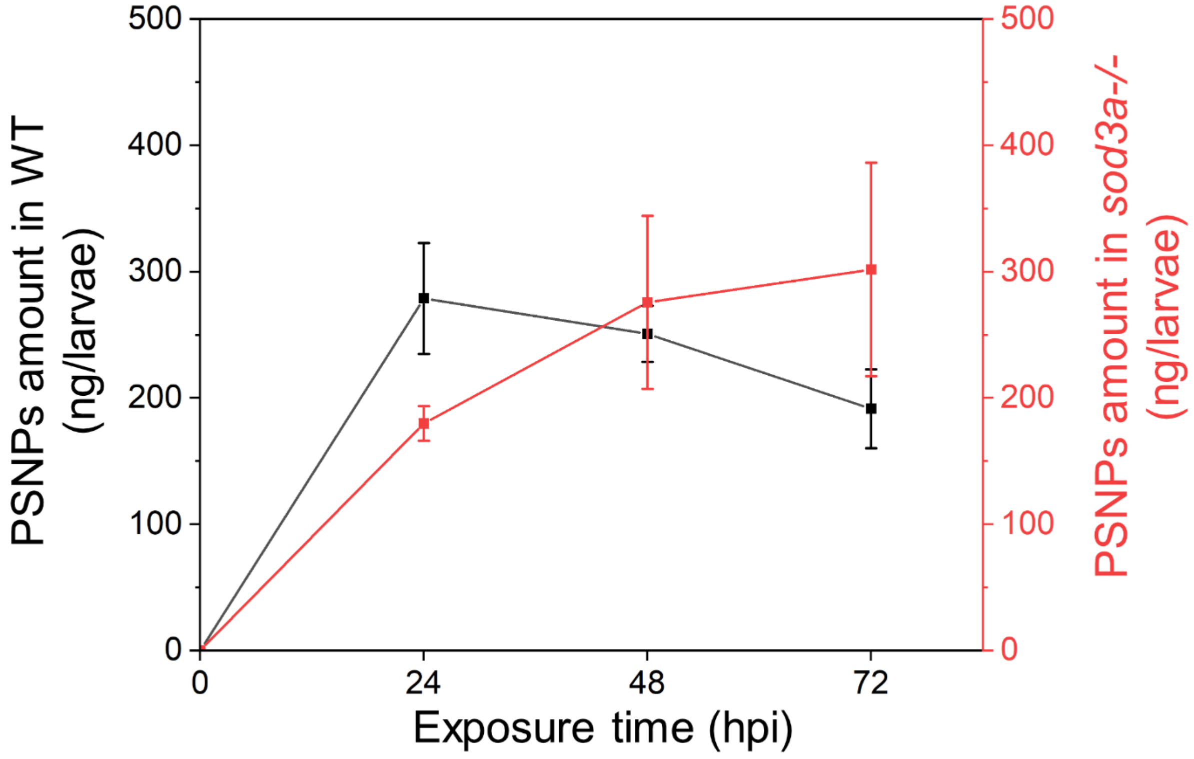

Fig. S3. PSNPs amounts in the whole body of WT and *sod3a*-/- zebrafish larvae exposed to PSNPs (50 μg/mL) from 4 to 7 dpf, measured at 24, 48, and 72 hpi.

Reference

[1] Karra, R., Knecht, A. K., Kikuchi, K., & Poss, K. D. (2015). Myocardial NF-κB activation is essential for zebrafish heart regeneration. Proceedings of the National Academy of Sciences, 112(43), 13255-13260.
